# Metabolic responses to polychromatic LED and OLED light at night

**DOI:** 10.1038/s41598-021-91828-6

**Published:** 2021-06-11

**Authors:** Asuka Ishihara, Insung Park, Yoko Suzuki, Katsuhiko Yajima, Huiyun Cui, Masashi Yanagisawa, Takeshi Sano, Junji Kido, Kumpei Tokuyama

**Affiliations:** 1 International Institute for Integrative Sleep Medicine (WPI-IIIS), University of Tsukuba, Tsukuba, Ibaraki Japan; 2 Ph.D. Program in Human Biology, School of Integrative Global Majors, University of Tsukuba, Tsukuba, Japan; 3 Faculty of Pharmaceutical Sciences, Josai University, Saitama, Japan; 4 Graduate School of Comprehensive Human Sciences, University of Tsukuba, Ibaraki, Japan; 5 Innovation Center for Organic Electronics, Yamagata University, Yamagata, Japan; 6 Graduate School of Organic Materials Science, Yamagata University, Yamagata, Japan

**Keywords:** Physiology, Metabolism

## Abstract

Light exposure at night has various implications for human health, but little is known about its effects on energy metabolism during subsequent sleep. We investigated the effects of polychromatic white light using conventional light-emitting diodes (LED) and an alternative light source, organic light-emitting diodes (OLED), producing reduced spectral content in the short wavelength of blue light (455 nm). Ten male participants were exposed to either LED, OLED (1000 lx), or dim (< 10 lx) light for 4 h before sleep in a metabolic chamber. Following OLED exposure, energy expenditure and core body temperature during sleep were significantly decreased (*p* < 0.001). Fat oxidation during sleep was significantly reduced (*p* = 0.001) after the exposure to LED compared with OLED. Following exposure to OLED, fat oxidation positively correlated with the 6-sulfatoxymelatonin levels, suggesting that the role of melatonin in lipolysis differs depending on the light. These findings advance our knowledge regarding the role of light in energy metabolism during sleep and provide a potential alternative to mitigate the negative consequences of light exposure at night.

## Introduction

Artificial light at night has transformed the lives of human beings, enabling a wide array of evening activities. Accompanied by this development in the modern-day world and lifestyle dominated by electronic devices, extended exposure to light during the dark hours has also brought negative consequences on human health, including alterations in sleep/wake regulation^1^, circadian rhythm^2^, thermoregulation^3^, and secretion pattern of hormones such as melatonin and cortisol^4^.

Light-emitting diodes (LED) are the most widely adopted lights for its advantages in easy usage, low power consumption, long lifetime, and instant ignition at low temperatures, but their emission spectrum is rich in the short wavelength of blue light at 460 nm^5^. A previous study demonstrated that the exposure to monochromatic blue light (469 nm) between 2:00 and 3:30 elicited an increase in plasma melatonin suppression compared to fluorescent white light in a dose dependent manner^6^. In addition, light-emitting electronic devices 4 h prior to sleep promotes alertness by decreasing delta/theta activity, suppressing melatonin, and increasing sleep latency^7^. The use of alternative light sources has been proposed to mitigate the negative physiological effects of LED. Organic light-emitting diodes (OLED) have recently gained popularity, especially for use in displays and devices for their glare-free, flexible nature, as well as its ability to achieve color temperature as low as 1773K^8^, but most importantly for its spectral content of polychromatic white light containing less blue light^9^.

The difference in the spectral composition between LEDs and OLEDs is crucial because melanopsin-expressing intrinsically photosensitive retinal ganglion cells (ipRGCs) are most sensitive to short wavelength of blue light^10^. These non-image-forming photoreceptors transmit their signals to the suprachiasmatic nucleus in the anterior hypothalamus, thereby entraining the circadian rhythm, and further project information to the pineal gland where they affect melatonin synthesis^11^. Park and colleagues demonstrated that the delay in dim light melatonin onset (DLMO) was greater by exposure to LED light 6.5 h prior to sleep compared to the delay in DLMO by OLED. However, studies evaluating the effects of OLED have been scarce to fully understand its impact on human physiology.

In addition, the rapid development and increase in the number of studies of artificial light at night have provided insight into its important role in metabolic health. The results of both animal model and epidemiologic studies have demonstrated a link between light exposure and an increased risk of weight gain^13^, obesity^14–16^, insulin resistance^17^, and metabolic impairments^18^. An emerging topic of interest is the effects of light, particularly OLED, on energy expenditure and substrate oxidation. Morning light exposure as a treatment for individuals with seasonal depression lowers^19^ or has no significant immediate effect on resting metabolic rate^20^. Daytime light treatment with 2500 lx between 14:00 and 16:00 for 1 week increases oxygen consumption in individuals with seasonal depression^21^. Daytime light exposure at 750 lx for 14 continuous hours starting at 08:00 in healthy participants has no significant impact on 24-h energy expenditure or substrate oxidation^22^. Exposure to monochromatic blue light (465 nm) 2 h before sleep does not affect energy metabolism during sleep, whereas it decreases energy expenditure, oxygen consumption, and carbon dioxide production after waking the next morning^23^. Thus, the impact of light exposure at night on energy metabolism during sleep remains unknown.

Here, we aimed to examine the effects of exposure to light with varying spectral composition on energy metabolism and sleep using polychromatic OLED and LED lights. We hypothesized that OLED containing less blue light relative to LED would have minimal effects on sleep and energy metabolism, comparable to that of dim light condition. Administration of light was set to evening to assess the immediate effects of light exposure on energy metabolism during sleep. With the rapid development and widespread use of light at night in our current society, understanding the physiological effects of these lights is crucial when considering long-term implications.

## Results

### Sleep

The overall sleep architecture did not differ significantly among LED, OLED or dim light conditions (Table 1). To investigate the effects of light exposure on sleep homeostasis, we analyzed the EEG delta power (0.5–4 Hz) during slow-wave sleep. Neither the delta power time course (*p* = 0.125; Supplementary Fig. 1a) nor the delta power density (*p* = 0.425; Supplementary Fig. 1b) differed significantly among the conditions. Subjective sleep and sleepiness assessment scores using the Oguri-Shirakawa-Azumi sleep inventory MA version and Karolinska Sleepiness Scale also did not differ significantly among the conditions (Supplementary Table 1, Supplementary Fig. 2).

**Table 1:** Sleep architecture.

	Dim	LED	OLED	p^†^
TIB	420	420	420	
TST	387.7 ± 9.7	396.6 ± 3.5	384.7 ± 8.2	0.481
Sleep latency	4.8 ± 1.7	5.7 ± 2.4	14.5 ± 8.4	0.281
SWS latency	21.6 ± 3.0	20.3 ± 4.3	30.4 ± 9.6	0.233
REM latency	120.8 ± 16.8	99.1 ± 14.2	109.7 ± 23.2	0.639
N1	45.9 ± 4.6	47.6 ± 6.8	44.8 ± 5.0	0.786
N2	199.6 ± 11.7	208.6 ± 13.1	194.7 ± 11.7	0.392
SWS	68.7 ± 9.0	66.6 ± 10.7	73.9 ± 7.9	0.496
REM	73.6 ± 9.2	73.9 ± 7.7	71.4 ± 6.8	0.870
WASO	27.9 ± 9.2	19.6 ± 2.2	21.2 ± 4.0	0.495
SE (%)	92.3 ± 2.3	94.4 ± 0.8	91.6 ± 1.9	0.481

Sleep parameters indicated as TIB, Time in bed; TST, total sleep time; SWS, slow-wave sleep; REM, rapid eye movement; N1, non-rapid eye movement sleep stage 1; N2, non-rapid eye movement sleep stage 2; WASO, wake after sleep onset; SE, sleep efficiency. Values are mean ± SE in minutes (*n* = 10). *p*^†^; one-way repeated measures ANOVA.

### Energy metabolism

**Figure 1:**
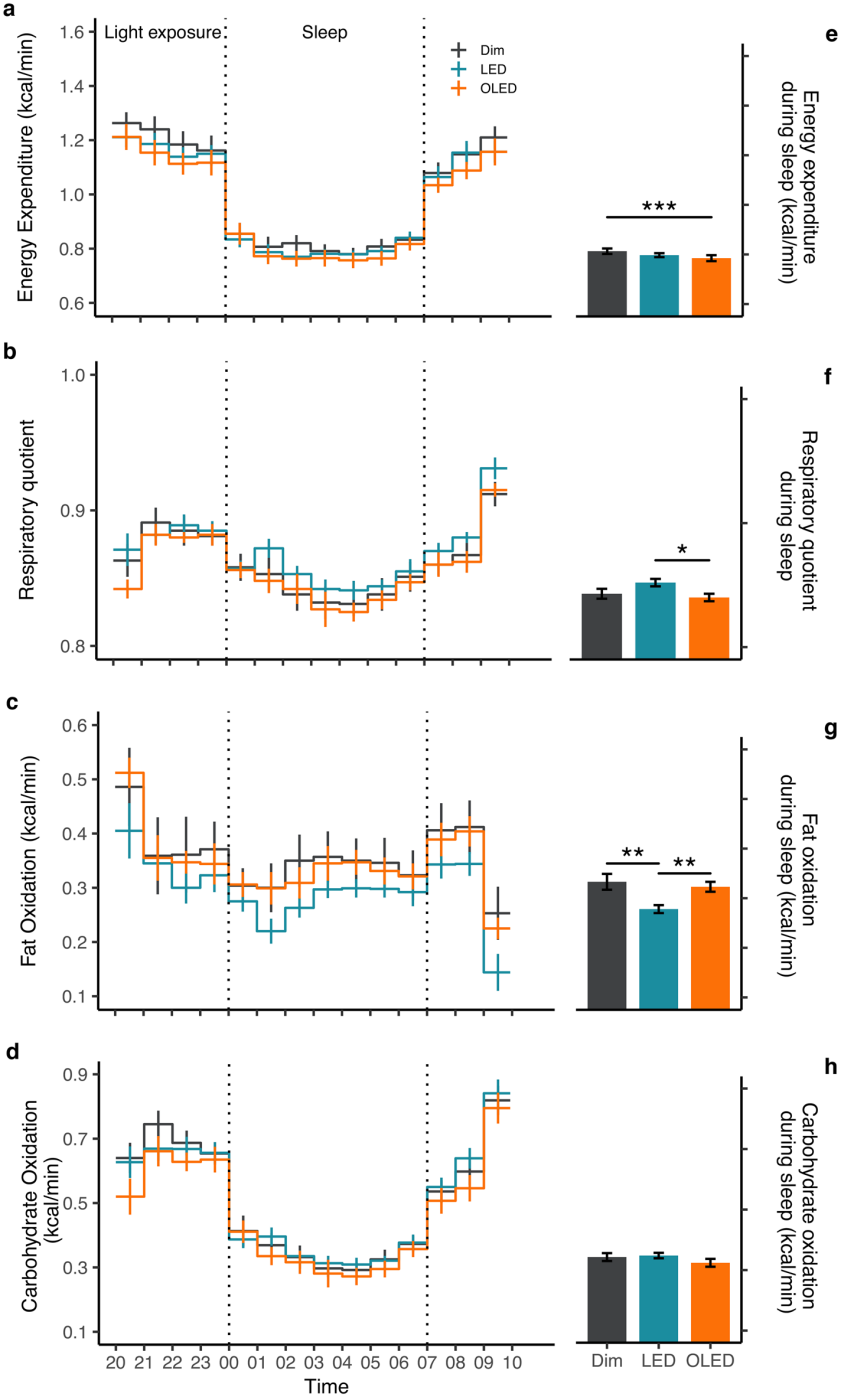
Energy metabolism. Time course (left panel) and mean values during sleep (right panel) are shown for (**a**) energy expenditure, (**b**) respiratory quotient, (**c**) fat oxidation, and (**d**) carbohydrate oxidation indicated as mean ± SE (*n* = 10). Significant differences among light conditions in energy metabolism during sleep were assessed by one-way repeated measures ANOVA with Bonferroni’s adjustment, *p < 0.05, **p < 0.01, ***p < 0.001.

Mean energy expenditure, respiratory quotient (RQ), and fat oxidation were analyzed separately according to sleep and wake periods (Fig. 1, Supplementary Fig. 3). During sleep, there was a difference in mean energy expenditure (F_2,138_ = 6.1, *p* = 0.003) with a significant post hoc comparison between OLED and dim light (p < 0.001; Fig. 1e). Mean RQ during sleep was also different among the light conditions (F_1,126_ = 3.8, *p* = 0.029) with significant post hoc comparison between LED and OLED (*p* = 0.016; Fig. 1f). This was consistent to the decrease in fat oxidation (F_1,111_ = 6.8, *p* = 0.003) with post hoc comparison revealing significance between LED and OLED (*p* = 0.001), and LED and dim light (*p* = 0.003; Fig. 1g). The effect of light persisted to the next morning after waking (Supplementary Fig. 3). Differences in mean energy expenditure (F_1,63_ = 7.5, *p* = 0.002) indicated significant post hoc comparison between dim light and OLED (*p* = 0.001), and dim light and LED (*p* = 0.047) (Supplementary Fig. 3a). Mean RQ remained high after waking (F_2,78_ = 3.7, *p* = 0.03) with a significant post hoc between LED and dim light (*p* = 0.024). Fat oxidation was significantly decreased (F_2,78_ = 5.6, *p* = 0.005) with post hoc comparison resulting in a significance between LED and dim light (*p* = 0.003; Supplementary Fig. 3b, c). Carbohydrate (*p* = 0.163) and protein oxidation (*p* = 0.307) were unaffected by the light conditions, both during sleep and after waking (Fig. 1h, Supplementary Fig. 3d). A two-way repeated measures ANOVA on the time course of energy metabolism with factors of light condition and time revealed no significant main effect of light condition or of the interaction between condition and time (Fig. 1).

### Thermoregulation

**Figure 2:**
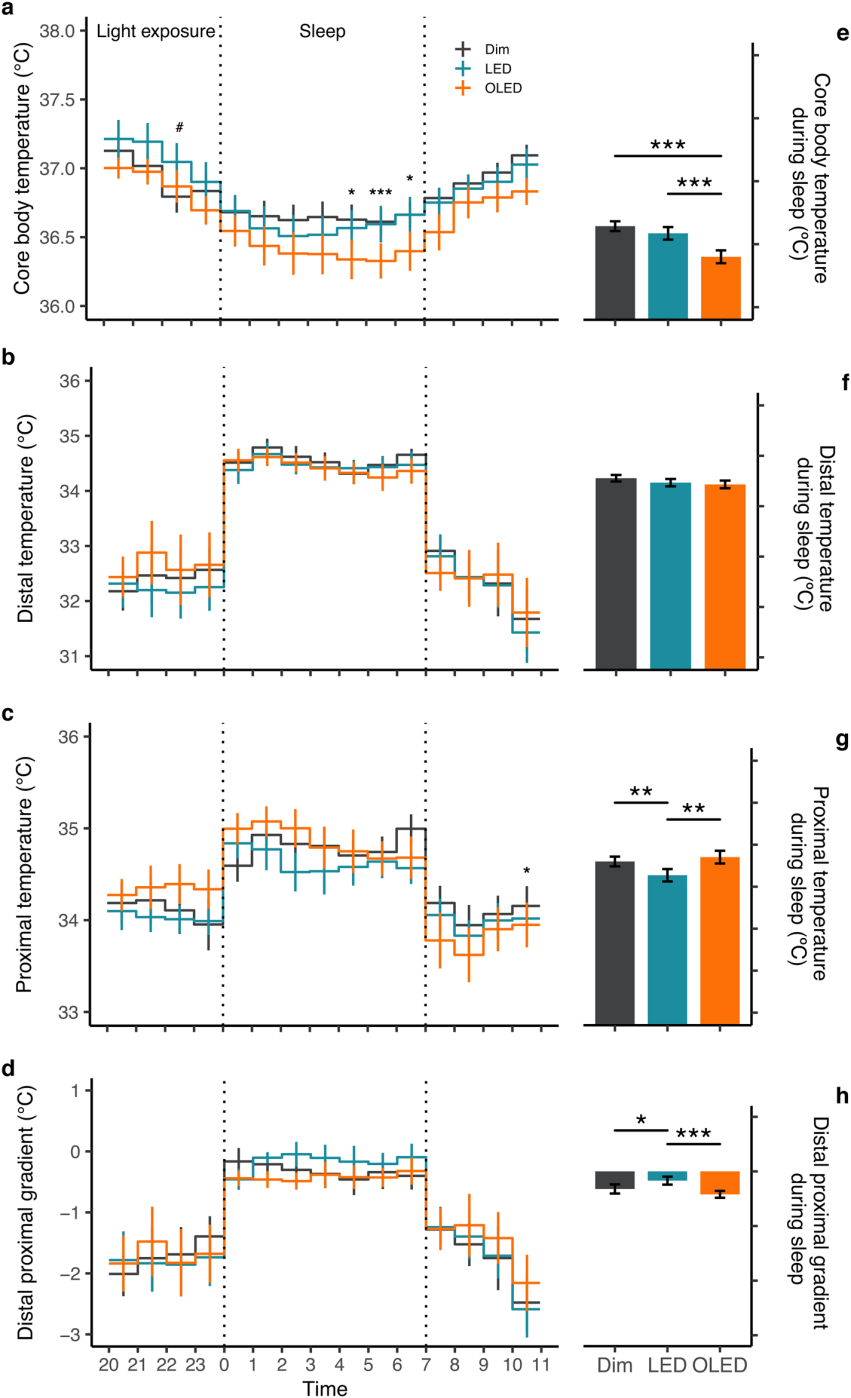
Thermoregulatory measures. Time course (left panel) and mean values during sleep (right panel) are illustrated. (**a**) Core body temperature, (**b**) distal temperature, (**c**) proximal temperature, and (**d**) distal proximal gradient expressed as mean ± SE. Time course analysis from two-way repeated measures ANOVA, post-hoc pairwise comparison with Bonferroni’s adjustment; *p < 0.05, ***p < 0.001 between OLED and dim light, #*p* < 0.05 between LED and dim light. Mean temperature during sleep by light condition from one-way repeated measures ANOVA, post-hoc pairwise comparison with Bonferroni’s adjustment; *p < 0.05, **p < 0.01, ***p < 0.001. (*n* = 10 core body temperature; *n* = 8 skin temperature).

A two-way repeated measures ANOVA revealed no significant effect of the light condition on the time course of the core body temperature (*p* = 0.162), but a significant interaction between condition and time (F_30,250_ = 1.7, *p* = 0.016; Fig. 2a). Post hoc analysis showed a significant increase in body temperature in LED compared with dim light before sleep and a significant decrease in OLED compared with dim light during sleep (Fig. 2a). Mean body temperature was significantly lower (F_1,120_ = 21.1, *p* < 0.001) with post hoc revealing significant difference between OLED and dim light, and OLED and LED, both during sleep (*p* = 0.001; Fig. 2e) and after waking (*p* < 0.001; Supplementary Fig. 4a).

Skin temperature was assessed on the basis of proximal and distal temperatures from a total of eight locations. A two-way repeated measures ANOVA on the time course of the proximal temperature revealed no significant effect of the light condition (*p* = 0.327), but a significant interaction between condition and time (F_30,201_ = 1.8, *p* = 0.009). Post hoc analysis indicated a significant decrease in OLED compared with dim light after waking at 10:00 (Fig. 2c). Furthermore, proximal temperature, assessed at the forehead demonstrated a significant increase in OLED compared with LED during and after sleep, but no significant differences were observed in other locations (Supplementary Fig. 5). No significant main effects of light condition were detected for distal temperature or the distal proximal gradient (DPG); nor was there any interaction between condition and time.

Mean temperature during sleep was then assessed using a one-way repeated measures ANOVA on distal and proximal temperatures and on the DPG (Fig. 2). During sleep, the proximal temperature was different among the conditions (F_2,96_ = 8.6, *p* = 0.0004) with significant post hoc comparison between LED and OLED (*p* = 0.004), and LED and dim light (*p* = 0.002) (Fig. 2c). A greater widening of the DPG (F_2,96_ = 7.2, *p* = 0.001) was observed in both dim light (*p* = 0.02) and OLED (*p* < 0.001) compared with LED after post hoc analysis (Fig. 2h). The distal temperature did not differ significantly among the light conditions (Fig. 2f).

**Figure 3:**
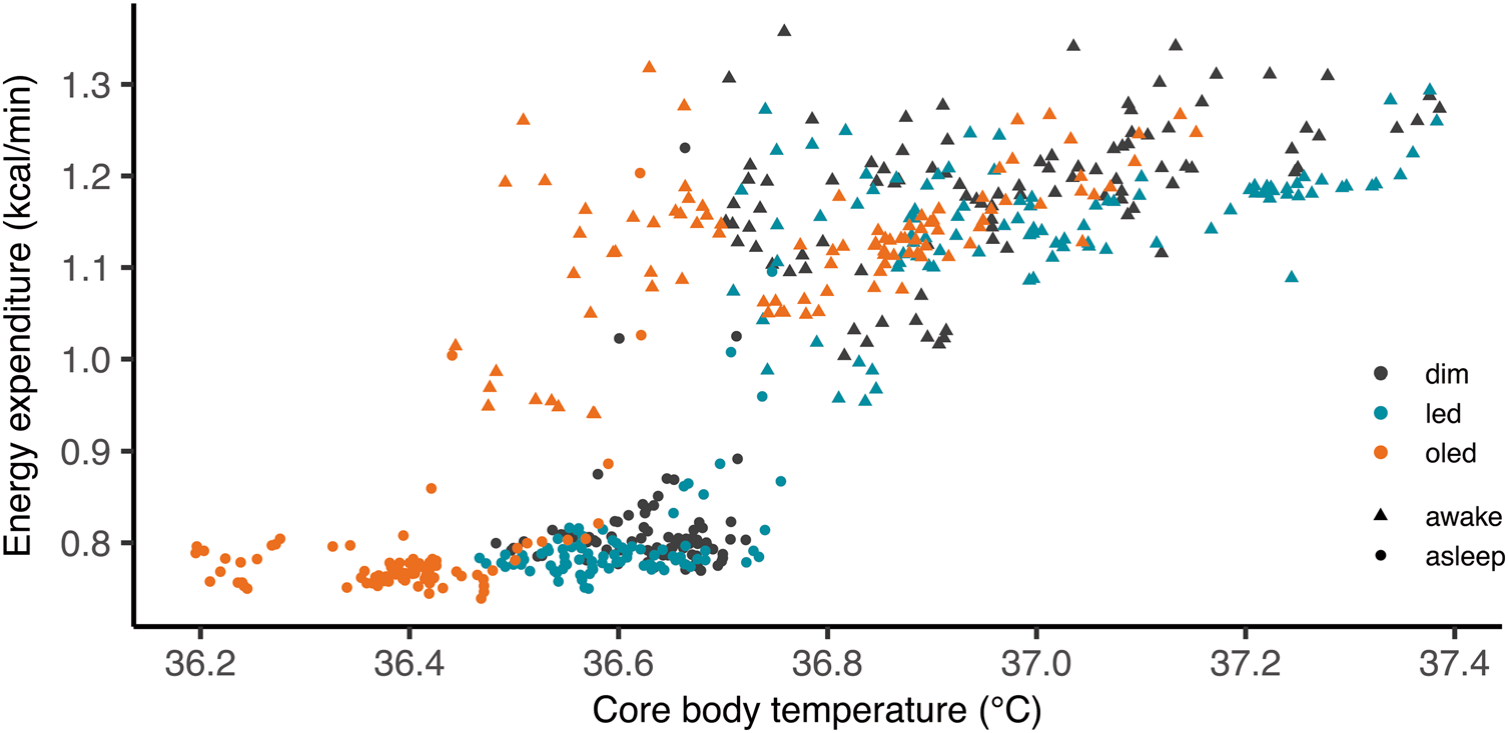
Correlation between core body temperature and energy expenditure. Correlation plotted as 5-min mean during sleep (circle) and wake (triangle) for dim (black), LED (blue), and OLED (orange) conditions.

Relationship between body temperature and energy expenditure during the sleep and wake periods are shown in Fig. 3. In all lighting conditions, core body temperature and energy expenditure showed a higher value during wake period compared to that of sleep period (Fig. 3).

### Urinary melatonin

**Figure 4:**
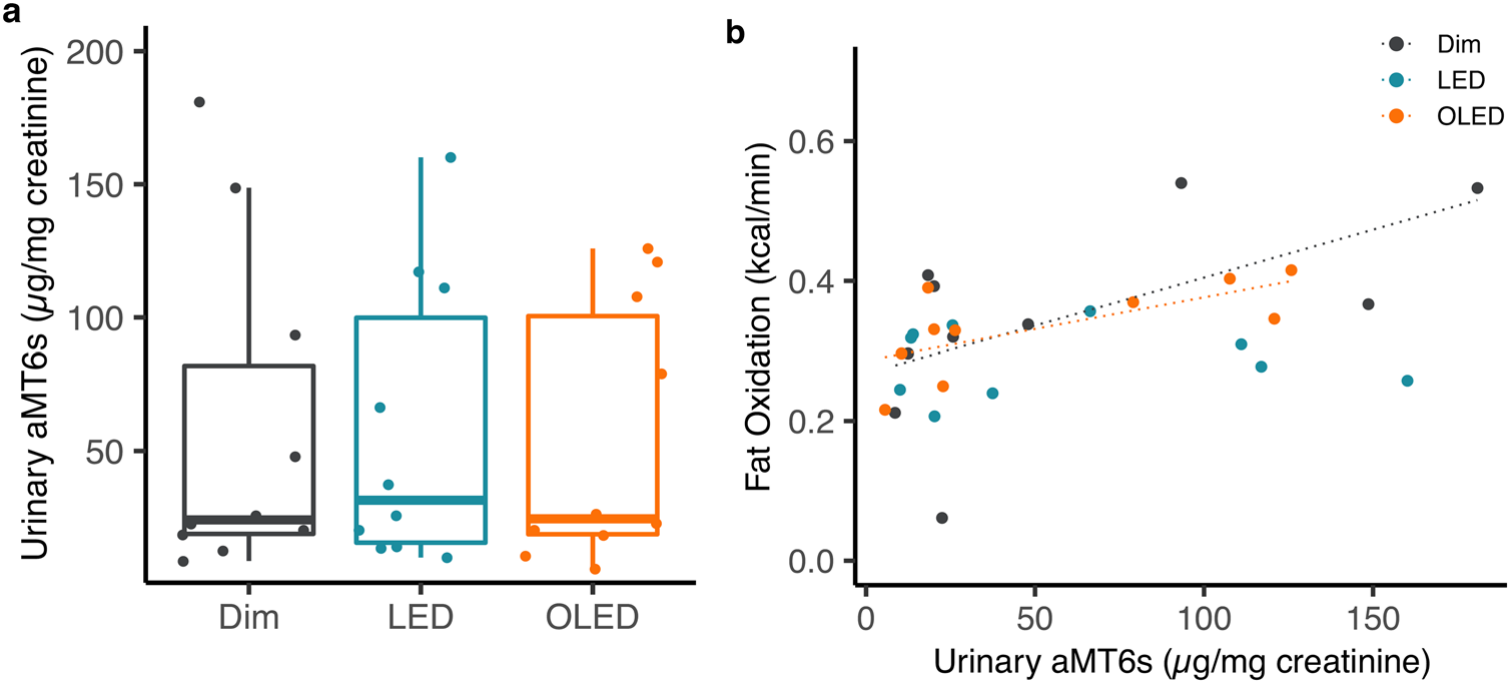
Urinary 6-sulfatoxymelatonin (aMT6s). (**a**) Concentration of total aMT6s normalized to urinary creatinine concentration. (**b**) Correlation between urinary aMT6s and fat oxidation of dim (black), LED (blue), and OLED (orange) light.

Total urinary excretion of 6-sulfatoxymelatonin (aMT6s) did not differ between the light conditions (*p* = 0.923; Fig. 4a). Urinary melatonin metabolites and energy metabolism were then further analyzed stratified by light conditions. Urinary aMT6s positively correlated with energy expenditure under OLED (Supplementary Fig. 6). Urinary melatonin metabolites showed no correlation with RQ in each of the light conditions; dim light (*p* = 0.52), OLED (*p* = 0.42), and LED (*p* = 0.41; Supplementary Fig. 6). A significant positive correlation between urinary aMT6s and fat oxidation was observed under OLED (r^2^ = 0.46, *p* = 0.032). There was no correlation under dim light (r^2^ = 0.36, *p* = 0.068) or LED (*p* = 0.96; Fig. 4b).

## Discussion

Evening exposure to polychromatic light altered the energy metabolism and core body temperature during sleep while showing little effect on the sleep architecture, suggesting that the influence of light on energy metabolism and thermoregulation observed in the present study is possibly affected by regulatory systems unrelated to those of sleep. The effect on metabolism continued until after waking the subsequent morning.

Wavelength dependency of light on sleep and the circadian rhythm is such that exposure to shorter wavelength of light in the evening suppress melatonin and increase sleep latency^24^. Exposure to monochromatic blue light (460 nm) compared to green light (550 nm) reduces slow wave-activity^24^, and polychromatic blue-enriched light reduces frontal NREM slow-wave activity during the first cycle of sleep^25^. In the present study, however, differences in homeostatic sleep pressure were not observed from a one-night exposure to spectrally different light, but this was consistent with findings from a previous study comparing the effects of OLED and LED on sleep^12^. The contradictory results may be due to the methods of administering light among the studies. Direct, high-intensity light utilizing a customized ganzfeld dome or goggles is often used in light exposure experiments^26,27^. In the present study, as well as in the previous study by Park and colleagues^12^, light exposure was conducted using ambient light placed above or in front of the participant’s angle of gaze. Thus, the precise amount of light reaching the retina at the cornea level remains to be an approximation. Nevertheless, it should be noted that ambient lighting more closely resembles our daily light exposure.

The alerting effect of light before sleep is reflected in EEG activity^7^ but also on thermoregulatory parameters such that exposure to bright light prior to sleep significantly increases core body temperature compared with dim light^1,28^. Likewise, evening exposure to monochromatic blue light significantly increases the core body temperature compared with monochromatic green and dim light^3,24^. The significant increase in core body temperature in the LED condition before sleep in the present study also supports the alerting effect of high-content blue light that was evaluated in previous studies.

Core body temperature is known to decrease before and during sleep, reflecting the suppression of heat production and enhancement of heat loss from distal regions of the body^29^. Heat dissipation from the core to the periphery is described as DPG, and its increase is associated with sleep propensity^30^. In the present study, despite the significant decrease in core body temperature during sleep in OLED compared with dim light, DPG and the proximal/distal temperatures did not differ significantly between the two conditions. This is in line with a previous study reporting the inconsistency in heat production and heat dissipation following evening exposure to monochromatic blue, green, and dim light^3^. Therefore, heat dissipation alone is insufficient to explain the drop in the core body temperature during sleep exhibited in the present study in the OLED condition. Heat production in the form of energy expenditure was also significantly lower in OLED compared with dim light. This attenuation in energy expenditure, as well as core body temperature, may be the result of the Q_10_ effect (Q_10_ is a measure of the change in a biological process due to a change in temperature), which downregulates metabolism by decreasing body temperature. A previous study on patients with pathological conditions showed that a 1ºC increase in body temperature is associated with an approximate 13% increase in the metabolic rate^31^. The Q_10_ of biological reactions mainly ranges between 2.0 and 3.0 with a 7%-12% increase in the rate of a chemical reaction from a 1ºC rise in temperature^32^. Because the regression analysis in the present study showed a 10.4% change in energy metabolism due to an increase of 1ºC, the decrease in energy expenditure in OLED may be explained by the Q_10_ effect. Body temperature, however, also varies with energy expended in the form of heat production fueled by protein, fat, and carbohydrates. Thus, the amount of macronutrient oxidized, ultimately contributing to thermoregulation, remains to be determined. Further studies are necessary to understand the causal relationship between energy expenditure and thermoregulation resulting from light exposure.

The ability to select fuel in response to alterations in the nutritional and physiological states is referred to as metabolic flexibility and is often measured as the change in RQ of whole-body energy metabolism^33^. During the fasting state, such as during sleep, energy metabolism becomes reliant on fat oxidation to maintain a low RQ^34^. The inability to switch to fat oxidation during sleep is associated with an increased risk of obesity^35^. In the present study, evening exposure to LED light resulted in a significantly higher RQ compared with exposure to the OLED light, and a significant decrease in fat oxidation during sleep. The difference in RQ during sleep suggests that exposure to different light spectra at night affects substrate oxidation, providing a plausible link between light exposure at night and weight gain^36^.

The specific factors that contribute to the alteration of substrate oxidation by light exposure remain to be identified. One of the main key factors transmitting photic stimulation to regulate energy metabolism is the hormone melatonin, the secretion of which is suppressed by light with an optimal sensitivity to short wavelengths between 446 and 477 nm^26^. Melatonin, produced in the pineal gland, binds to melatonin receptors which are expressed throughout the body, including pancreatic islets, adipose tissue, skeletal muscle, and liver, thus entraining the downstream circadian rhythm^37,38^. In the present study, we assessed the concentration of aMT6s, the major urinary metabolite of melatonin, which is strongly correlated with the serum melatonin concentration^39^. The total excretion of aMT6s, however, did not differ among the light conditions. This is partly due to the poor time resolution of the collected urinary samples as they reflect the total, combined amount of melatonin metabolites from day 1 to day 2 of the experimental days while participants were in the metabolic chamber. For this reason, temporal changes in the concentration of melatonin metabolites were not identified. Additionally, because melatonin has a large inter-individual variability^40^, we further analyzed the correlation between aMT6s and metabolic parameters. Our results indicated that aMT6s excretion was marginally (*p* = 0.068) and significantly (*p* = 0.032) correlated with fat oxidation in the dim and OLED conditions, respectively. This finding was consistent with that of a previous study showing an inverse correlation between RQ and melatonin^41^. Interestingly, the tendency toward a positive correlation between aMT6s excretion and fat oxidation was not observed in the LED condition (*p* = 0.96). This suggests that the role of melatonin to stimulate lipolysis in intramuscular adipocytes observed in porcine and bovine^42,43^ may not be preserved under exposure to LED, but retained under exposure to OLED, possibly due to the reduction in the blue light spectrum. However, since OLED showed a stronger positive correlation compared to dim light despite the increase in intensity and short wavelength of blue light, spectral composition alone seems to be insufficient to explicate these results. Temporal changes in melatonin concentration need to be assessed to further understand its relation to energy metabolism during sleep. Additionally, although the spectral peak occurred at 455 nm for LED, limitations exist in using blue light to excite melanopsin with a maximum sensitivity at 480nm^44^. Considering that the melanopic lux between LED and OLED in the present study were 730 and 780 lx respectively, the effect of the spectral composition of LED peaking at 454 nm of blue light did not reflect clearly on the melanopic functions. In addition, since the non-image forming responses to light is partially compensated by rods and cones^45^, melanopic responses alone may not be sufficient to explicate the effects of spectral differences between LED and OLED.

In the present study, measurements of energy metabolism and thermoregulation were conducted for only one night, thereby limiting our understanding of the overall metabolic changes throughout the day. Considering that the physiological impact of light varies with the timing of the exposure^46^, it is likely that daytime metabolism is altered depending on the intensity, duration, and wavelength of light. Additionally, although our experiment was not designed to account for the circadian changes caused by light exposure, a preliminary study showed that maintaining a modified constant posture in dim light (< 10 lx) for 8 h prior to sleep moved the core body temperature nadir time to either 30 min before or at sleep onset (unpublished data). Thus, it is important to note whether dim light is indeed an ideal control to compare with the other light exposure conditions as it has its own characteristic to possibly shift body temperature to an earlier time. Additionally, future studies involving light exposure must take into consideration the participants’ age and sex, as these factors may also impact energy metabolism^47,48^.

The present study is one of the first to show that evening light exposure affects metabolism by selectively utilizing substrates during sleep and the subsequent morning after waking. The contrasting metabolic outcomes observed in the LED and OLED conditions may indicate differences in the spectral composition of light in which the short wavelength of blue light negatively affects energy metabolism by increasing the RQ and decreasing fat oxidation during sleep and after waking. Because the spectral composition and melanopic lux between LED and OLED did not differ greatly, it is important to note that characteristics of light, apart from wavelength, such as the glare, luminance, and frequency of fluctuation^49^, may play an additional role in human physiology. Nevertheless, these findings suggest that OLED may be a viable alternative source of light at night.

## Methods

### Participants

Ten healthy males (mean ± SE: 25.7 ± 0.65 years; BMI: 22.3 ± 0.65 kg/m^2^) participated in a balanced cross-over study. Participants were non-smokers, non-shift workers, and had not engaged in transmeridian travel within 1 month prior to the experiment day. Participants did not have major sleep disorders (Pittsburgh Sleep Quality Index score ≤ 5), did not take sleeping pills, and had intermediate chronotypes as assessed by the Morningness-Eveningness Questionnaire (MEQ)^50^. The study was conducted according to the guidelines of the Declaration of Helsinki and the ethics committee of the University of Tsukuba reviewed and approved this study (UMIN: 000042654). All participants signed an informed consent form before the experiment.

### Study protocol and light exposure

One week prior to the experiment day, the participants maintained a regular sleep and wake time and refrained from ingesting caffeine and alcoholic beverages for 3 days before the experiment day. They underwent an adaptation night prior to the experiment day to familiarize themselves with sleeping in the metabolic chamber room with polysomnographic recording. Body composition was measured using the bioimpedance method (BC-118E, TANITA, Tokyo, Japan).

**Figure 5:**
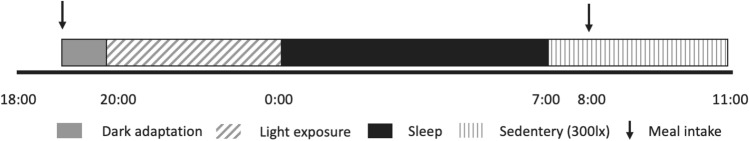
Study protocol. Exposure to either LED (1000 lx), OLED (1000 lx), or dim (< 10 lx) light at eye level for 4 h before sleep. Time of day indicated as relative hours with sleep time from 24:00 to 07:00.

On the experiment day, the participants arrived at the sleep laboratory 6 h prior to their habitual sleep time (Fig. 5). Relative clock time was normalized to a sleep time at 24:00 and wake time at 07:00. All sensors were attached before entering the metabolic chamber. Dinner was served after the 30-min dark adaptation time, which was conducted to attenuate any influence of daytime light exposure. The participants were then exposed to either the 1000 lx LED, 1000 lx OLED, or dim LED (< 10 lx) at eye level for 4 h continuously while maintaining a sitting posture until their habitual sleep time. They were prohibited from using any devices that emitted light. Hourly questionnaires using the Karolinska Sleepiness Scale and tasks were administered to assess sleepiness during light exposure. Immediately upon awakening, the Oguri-Shirakawa-Azumi sleep inventory middle-age version (OSA-MA) was given to evaluate their subjective sleep. The questionnaire assesses sleep on the basis of 5 factors, including sleepiness after waking, initiation and maintenance of sleep, dreaming, how refreshed they feel, and sleep length. Participants ate breakfast 1 h after waking, and subjective sleepiness and energy metabolism were continuously measured for 4 h after waking under regular room light (300 lx). Hourly questionnaires and tasks were administered as during the previous night.

**Figure 6:**
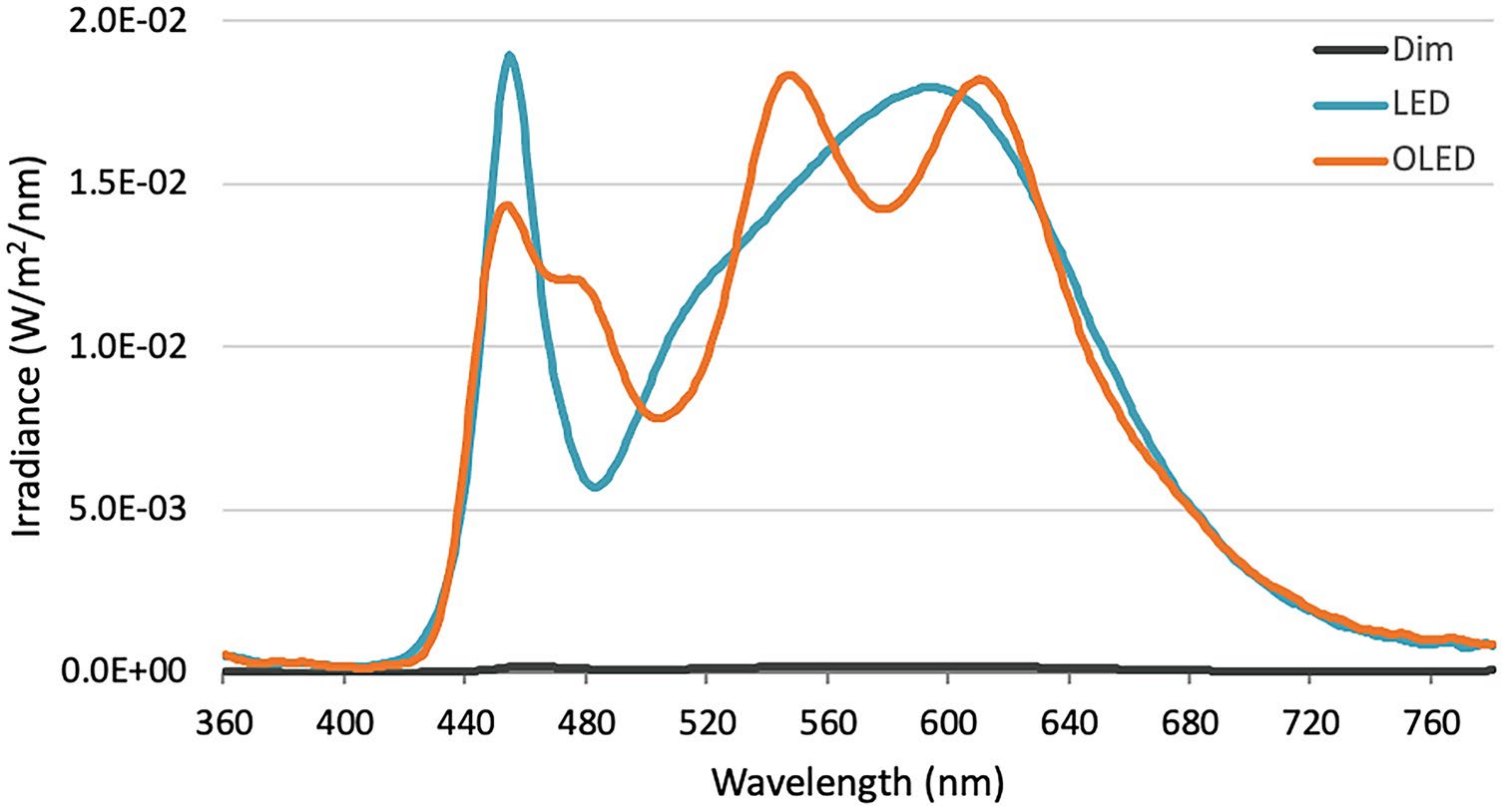
Spectral power distribution of LED, OLED, and dim lights expressed in irradiance.

Polychromatic white light exposure was conducted using LED (OL291241, ODELIC Co., Ltd., Japan) and OLED (P09, Lumiotec Inc., Japan). The spectral power distribution (Fig. 6) was measured using an illuminance spectrophotometer (CL-500A, Konica Minolta Inc., Tokyo, Japan). Spectral irradiance measured at 455 nm for LED was 1.89 × 10^−2^ W m^−2^^2^ nm^−1^ and OLED was 1.43 × 10^−2^ W m^−2^ nm^−1^ under the same color temperature 4000 K. Light panels were set against the wall (Organic Lighting Corporation, Yamagata, Japan) directly in front of the participants and adjusted to match the illuminance of around 1000 lx (log photon flux: 14.97 log_10_(cm^−2^ s^−1^)) at eye level with melanopic lux of 730 and 780 for LED and OLED respectively^51^. Illuminance was measured using a lux meter before the experiment (CL-70F, Konica Minolta Inc., Tokyo, Japan). Dim light and day-2 morning room light exposures were conducted using a ceiling-mounted LED (Kitera 100, Aurora Daiichi, Aichi, Japan).

### Measurements

#### Energy metabolism

A whole room metabolic chamber was used to measure energy metabolism (Fuji Medical Science Co., Ltd., Chiba, Japan). The airtight chamber, measuring 2.00 × 3.45 × 2.10 m with an internal volume of 14.49 m^3^, was furnished with a mattress, desk, chair, and toilet. Airflow in the chamber was ventilated at a rate of 80 L/min. Temperature and humidity were maintained at 25.0 ± 0.5 °C and 55.0 ± 3.0%, respectively. The oxygen (O_2_) and carbon dioxide (CO_2_) concentrations were measured by mass spectrometry (VG Prima δB, Thermo Electron Co., Winsford, UK). The precision of the mass spectrometry was calculated from the standard deviation of measurements of a calibrated gas mixture (O_2_ 15%, CO_2_ 5%), which was < 0.002% for both O_2_ and CO_2_. Hourly average O_2_ (V̇O_2_) consumption and CO_2_ production (V̇CO_2_) rates were calculated using an improved algorithm for transient responses^52^. Energy expenditure and macronutrient oxidation were calculated based on V̇O_2_, V̇CO_2_, and urinary nitrogen excretion^53^, and the RQ was determined as the ratio of V̇CO_2_ and V̇O_2_.

#### Sleep recordings and analysis

Sleep was recorded polysomnographically using a PSG-1100 (Nihon Kohden, Tokyo, Japan). Electrodes were attached to record the electroencephalograms (F3/M2, F4/M1, C3/M2, C4/M1, O1/M2, O2/M1), electrooculograms, and electromyograms. Sleep stages were visually scored by a registered polysomnographic technologist according to standard criteria^54^. Spectral analysis on C3/M2 was conducted using fast Fourier transformation on a 5-s window to obtain a 0.2 Hz resolution^55^. Delta power density was calculated during non-rapid eye movement sleep in the frequency range from 0.75 to 4.00 Hz for each 30-s epoch of sleep.

#### Thermometry

Core body temperature was continuously measured every 30 s using a CorTemp sensor and a data recorder (CorTemp, HQ Inc., Palmetto, FL, USA). The sensor in a single-use ingestible pill is accurate to ± 0.1ºC and was calibrated each time before use. Recorded data was reported as an hourly average.

Skin temperature was recorded at four proximal points; (infraclavicular area, midthigh on the right musculus rectus femoris, 1 cm above the navel on the stomach, and forehead) and four distal points; (back of right and left hands, middle of right and left foot instep). The mean proximal temperature was calculated with the following equation; (forehead × 0.093) + (thigh × 0.347) + (infraclavicular area × 0.266) + (stomach × 0.294). The mean distal temperature was the mean of both hands and feet. The DPG was calculated as the difference between the distal and proximal skin temperature^56^. Thermistor probes (ITP082-24, Nikkiso-Thermo Co., Tokyo, Japan) connected to a data logger (N543, Nikkiso-Thermo Co.) were used to continuously record the skin temperature.

#### Urinary 6-sulfatoxymelatonin assessment

Participants collected their urine samples continuously while they were in the metabolic chamber room from 4 h before sleep to 4 h after waking. Total volume was measured, and samples were stored at − 20ºC until assay. Urinary aMT6s was assayed from the sampled urine by a fluorometric high-pressure liquid chromatography^57^ comprised of an LC-20AD pump system (Shimadzu, Kyoto, Japan), equipped with RF-10-A spectrofluorometer (Shimadzu, Kyoto, Japan), Inertsil ODS-3 analytical column (5020-01732 GL Sciences, Tokyo, Japan), column oven kept at 40˚C (GL Science, Tokyo, Japan), and LCsolution (Version 1.22 SP1 software, Shimadzu, Kyoto, Japan). All aMT6s was assayed using indole-3-acetamide as the internal standard and normalized to urinary creatinine levels to control for variations in the urine concentration^58^.

### Statistics

All data are presented as mean ± SE. Time course data for delta power and energy metabolism were analyzed by two-way repeated-measures ANOVA. Data during sleep or the subsequent morning was compared using one-way repeated-measures ANOVA using post hoc Bonferroni’s adjustment for multiple comparisons. Greenhouse–Geisser correction was conducted when Mauchily’s spherecity was largely violated. Pearson’s correlation was conducted for the association analysis. Semiparametric regression analysis was used to analyze energy metabolism by sleep stages^59^. All statistical analysis was conducted using R studio (version: 1.2.1335, R Consortium, https://www.r-consortium.org).

## Supplementary Information

Supplementary Information.
