# Supplementary material for: Metabolic responses to polychromatic LED and OLED light at night
Source: Sci Rep. 2021 Jun 11;11:12402. doi: 10.1038/s41598-021-91828-6 (PMC8196130; doi:10.1038/s41598-021-91828-6)
Supplement: Supplementary file 1 — Supplementary Information. [file 41598_2021_91828_MOESM1_ESM.pdf]

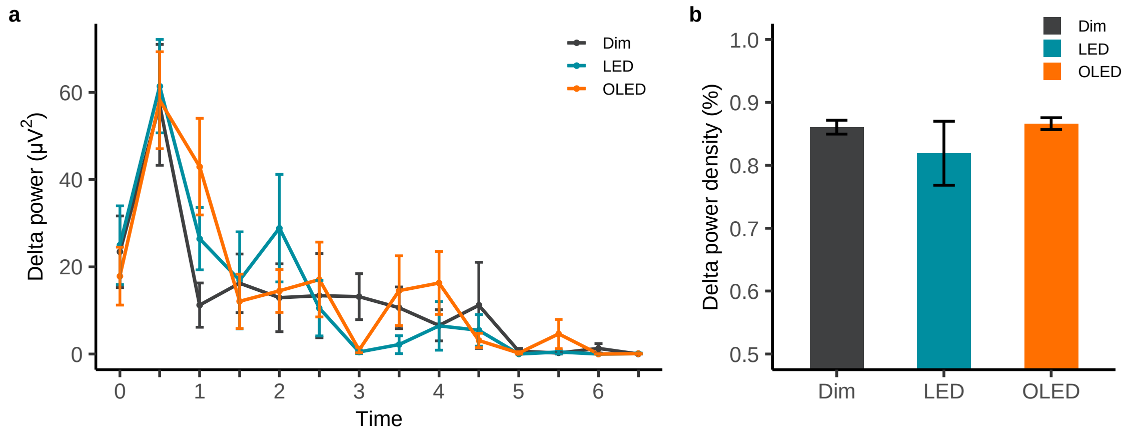

Supplementary Figure 1. Delta power during slow wave sleep. (**a**) Time course of delta power (mean ± SE) during SWS under dim (grey line), LED (blue line), OLED (orange line). (**b**) Delta power density expressed as the percentage of delta power over the total power (0.75-30.0Hz) during SWS under the three light conditions expressed as the mean ± SE (n=10).

Supplementary Table 1. Oguri-Shirakawa-Azumi (OSA) sleep questionnaire

	Dim	LED	OLED	$p^{\dagger}$
**Sleepiness**	47.0 ± 3.2	46.2 ± 3.2	47.0 ± 3.4	0.922
**Initiation and maintenance of sleep**	43.5 ± 1.5	41.9 ± 1.4	43.0 ± 1.9	0.712
**Frequent dreaming**	47.7 ± 4.6	47.2 ± 4.0	49.5 ± 4.4	0.916
**Refreshing**	48.8 ± 1.0	45.4 ± 2.7	44.8 ± 2.2	0.278
**Sleep length**	46.7 ± 2.7	43.0 ± 3.7	44.0 ± 3.8	0.537

Values represent the mean ± SE. $p^{\dagger}$; One-way repeated measures ANOVA (n=10).

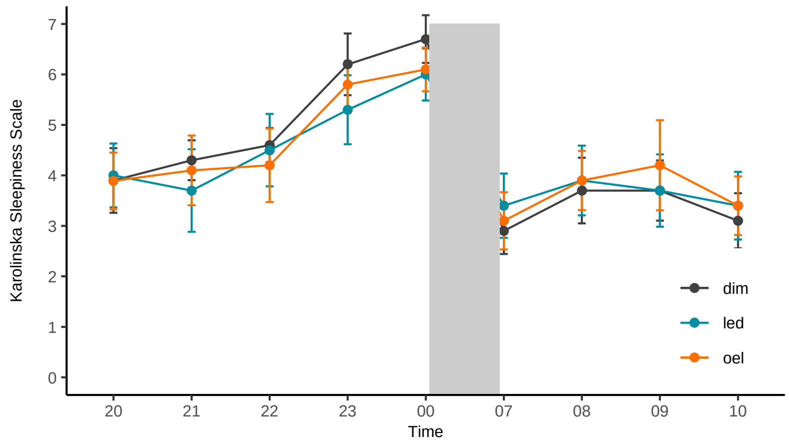

Supplementary Figure 2. Karolinska Sleepiness Scale (KSS). Hourly mean values of KSS scores (mean ± SE) before and after sleep for dim (black), LED (blue), and OLED (orange) (sleep time shaded in grey) (n=10).

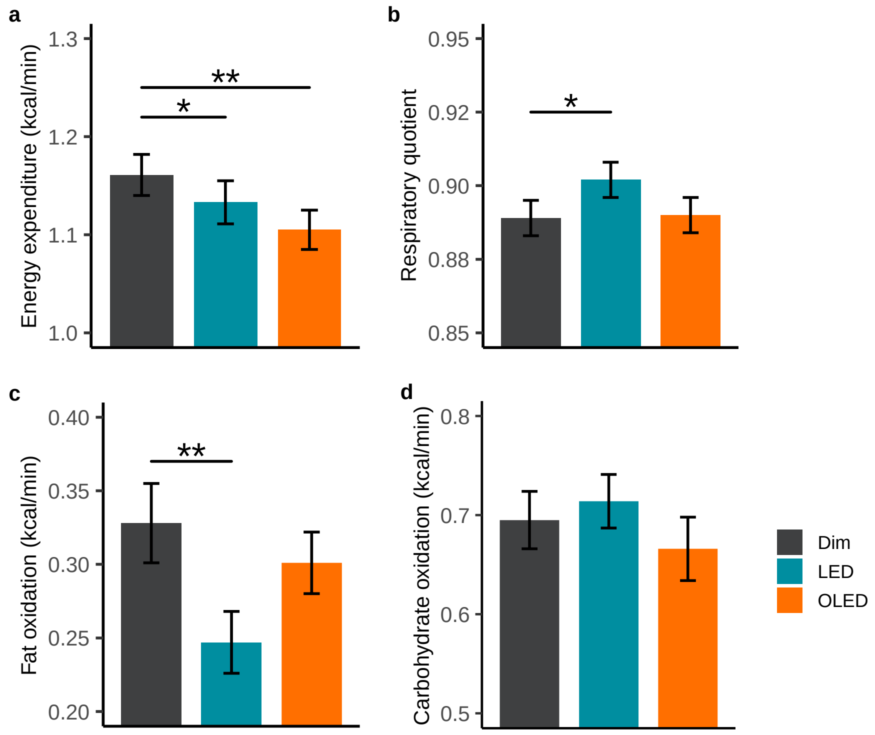

Supplementary Figure 3. Energy metabolism on day 2 after waking up. (a) Energy expenditure, (b) respiratory quotient, (c) fat oxidation, and (d) carbohydrate oxidation values provided as the mean ± SE; *p* values of one-way repeated measures ANOVA, $*p < 0.05$, $**p < 0.01$, $***p < 0.001$ (n=10).

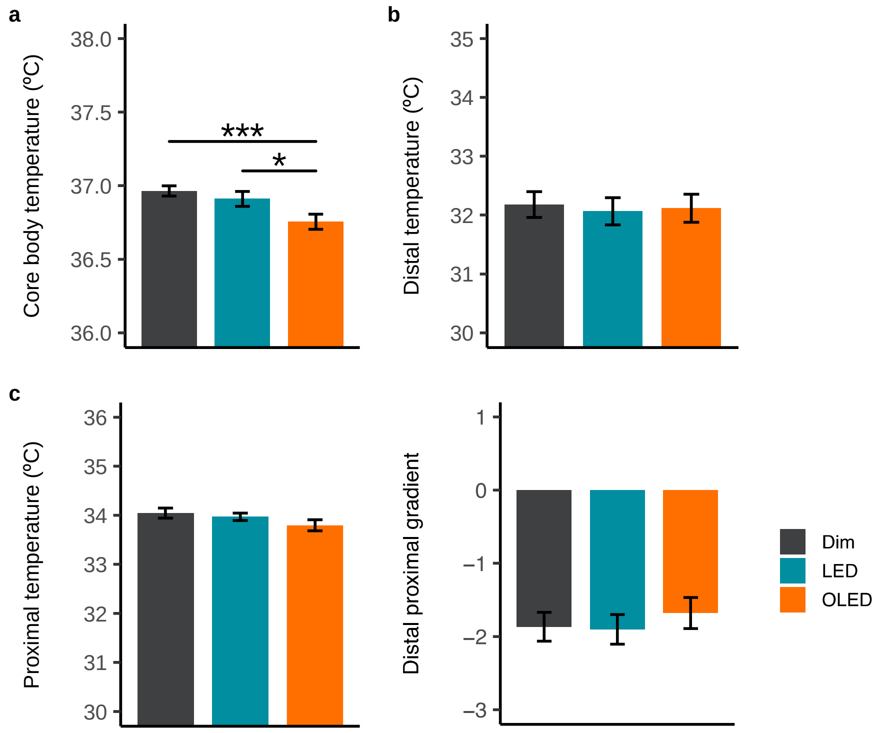

Supplementary Figure 4. Thermoregulatory measures on day 2 after waking up. (a) Core body temperature (n=10), (b) distal temperature, (c) proximal temperature, and (d) distal proximal gradient (DPG) values represented as the mean ± SE (n=8). *p* values of one-way repeated measures ANOVA with Bonferroni adjustment; *p <0.05, **p<0.01, ***p<0.001.

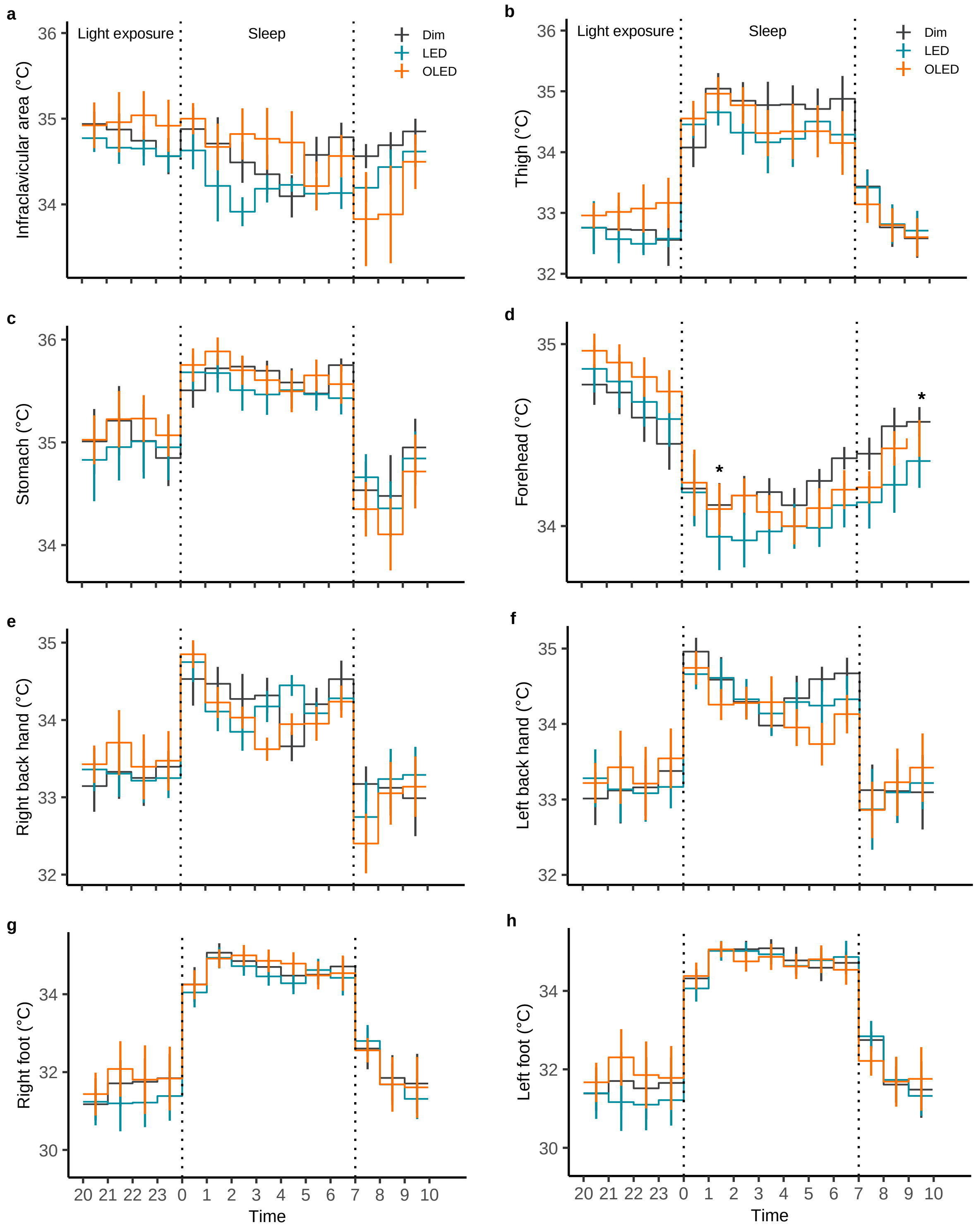

Supplementary Figure 5. Time course of skin temperature by locations. (a) Infraclavicular area, **(b)** thigh, **(c)** stomach, **(d)** forehead, **(e)** right back hand, **(f)** left back hand, **(g)** right foot, and **(h)** left foot temperature values provided as the mean ± SE (n=8) for dim (black), LED (blue), and OLED (orange). *p* values of two-way repeated measures ANOVA, *$p < 0.05$ between LED and OLED (n=8).

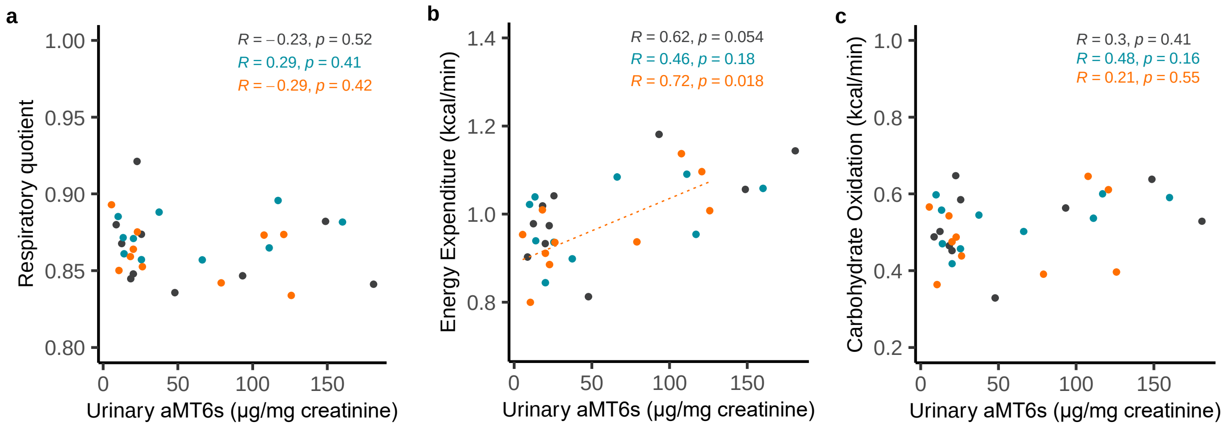

Supplementary Figure 6. Correlation between urinary aMT6s and metabolism. **(a)** Respiratory quotient, **(b)** energy expenditure, and **(c)** carbohydrate oxidation of dim (black), LED (blue), and OLED (orange) are shown. Pearson coefficient (R) and p-values are indicated, regression line representing significant correlation ($p<0.05$).
